# Data on the removal of fluoride from aqueous solutions using synthesized P/γ-Fe_2_O_3_ nanoparticles: A novel adsorbent

**DOI:** 10.1016/j.mex.2018.12.009

**Published:** 2018-12-18

**Authors:** Shahin Ahmadi, Somayeh Rahdar, Chinenye Adaobi Igwegbe, Abbas Rahdar, Nahid Shafighi, Fardin Sadeghfar

**Affiliations:** a Department of Environmental Health, Zabol University of Medical Sciences, Zabol, Iran; b Department of Chemical Engineering, Nnamdi Azikiwe University, Awka, Nigeria; c Department of Physics, University of Zabol, Zabol, P. O. Box. 35856-98613, Islamic, Iran

**Keywords:** Fluoride, P/γ-Fe_2_O_3_ nanoparticles, Aqueous solution, Isotherm, Kinetic

## Abstract

High concentration of fluoride above the optimum level can lead to dental and skeletal fluorosis. The data presents a method for its removal from fluoride-containing water. P/γ-Fe_2_O_3_ nanoparticles was applied as an adsorbent for the removal of fluoride ions from its aqueous solution. The structural properties of the P/γ-Fe_2_O_3_ nanoparticles before and after fluoride adsorption using the Fourier transform infrared (FTIR) technique were presented. The effects of pH (2–11), contact time (15–120 min), initial fluoride concentration (10–50 mg/L) and P/γ-Fe_2_O_3_ nanoparticles dosage (0.01–0.1 g/L) on the removal of F^−^ on P/γ-Fe_2_O_3_ nanoparticles were presented with their optimum conditions. Adsorption kinetics and isotherm data were provided. The models followed by the kinetic and isotherm data were also revealed in terms of their correlation coefficients (*R^2^*).

## Graphical abstract

**Figure:**
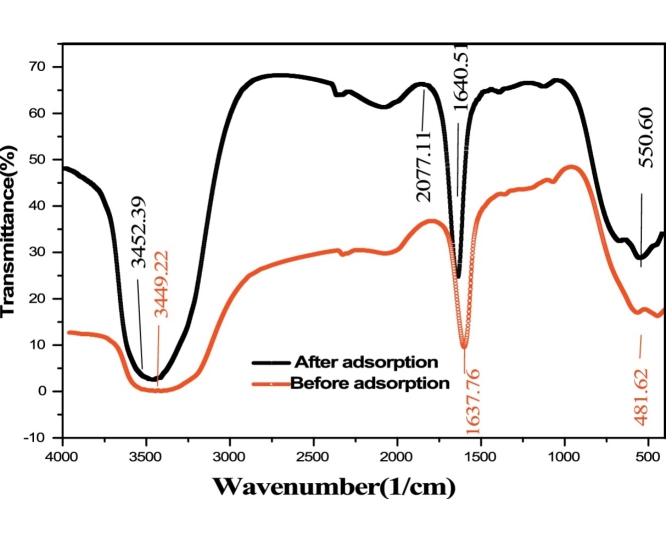

**Specifications Table**

**Table:** 

• Subject area	• Environmental Engineering
• More specific subject area	• Adsorption
• Type of data	• Image, table, and figure
• How data was acquired	• All adsorption experiments were done in batch mode. After the adsorption process, the residual fluoride concentrations were estimated. The initial and residual fluoride concentrations in the solutions were analyzed using a UV–visible recording spectrophotometer (Shimadzu Model, CE-1021-UK) at 570 nm. Fourier-transform infrared spectroscopy (FT-IR) was done on a JASCO 640 plus machine (in the range of 400-4000 cm^−1^) to determine the functional groups present in the adsorbent before and after fluoride adsorption. The pH of the solution was measured using a MIT65 pH meter.
• Data format	• Raw and analyzed
• Experimental factors	• The influence of pH, contact time, initial fluoride concentration and P/γ-Fe_2_O_3_ nanoparticles dosage on the adsorption process. Kinetic and isotherm parameters were also presented.
• Experimental features	• Fluoride removal from aqueous solution using P/γ-Fe_2_O_3_ nanoparticles. P/γ-Fe_2_O_3_ nanoparticle characterization data obtained from FTIR. Kinetic and isotherm modeling of the removal process.
• Trial registration	• Not applicable
• Ethics	• Not applicable

## Protocol data

• The presented data established that P/γ-Fe_2_O_3_ nanoparticles can be applied for the removal of fluoride with great efficiency.
• Data on the isotherm, kinetics, and effect of process variables were provided, which can be further explored for the design of a treatment plant for the treatment of fluoride-containing industrial effluents where a continuous removal is needed on a large scale.
• FTIR data for P/γ-Fe_2_O_3_ nanoparticles were also provided.
• The dataset will also serve as a reference material to any researcher in this field.

## Description of protocol

### Data

**Fig. 1:**
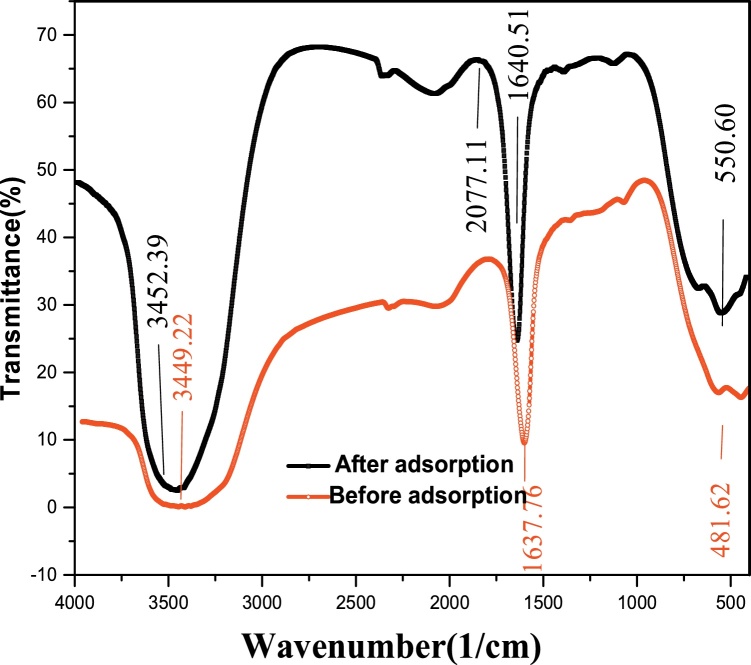
FTIR spectra of the P/γ-Fe_2_O_3_ nanoparticles before and after fluoride adsorption.

**Fig. 2:**
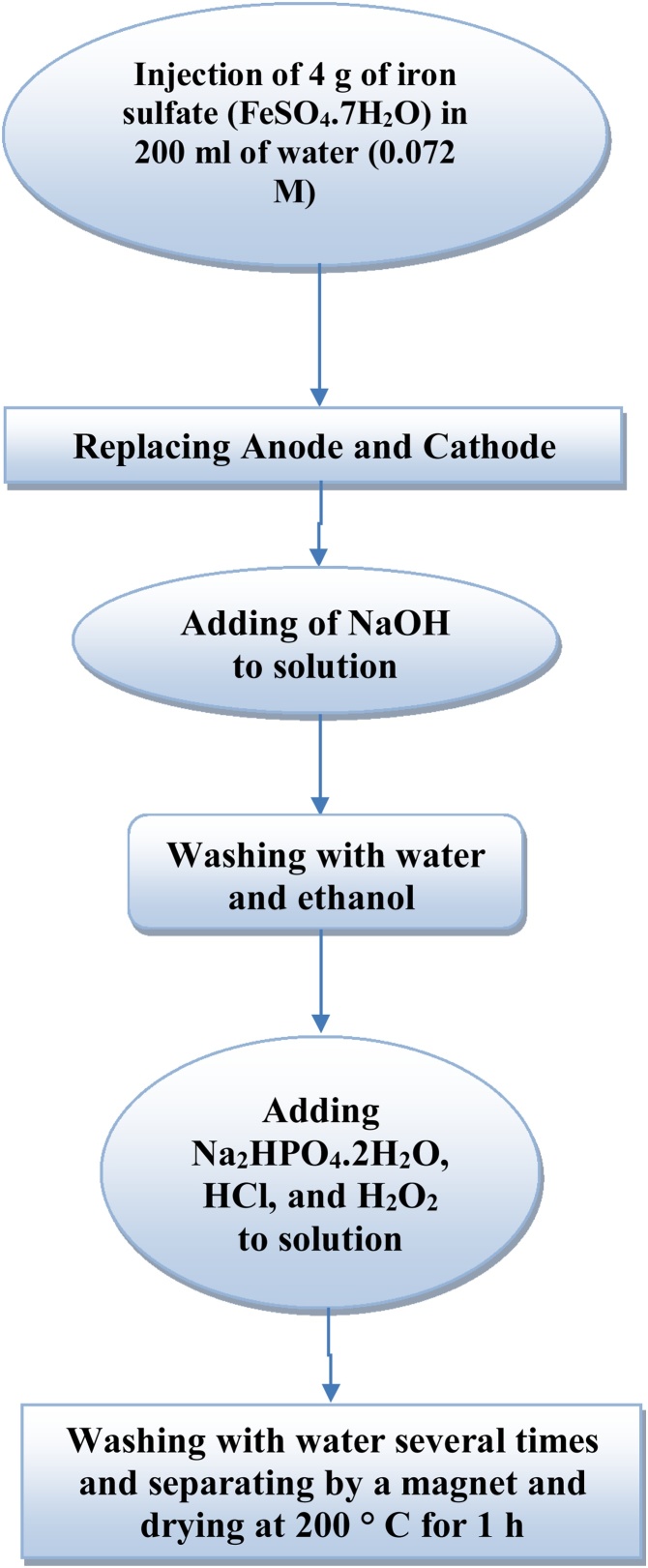
The schematic illustration of the synthesis of P/γ-Fe_2_O_3_ nanoparticles.

High concentration of fluoride is toxic and causes digestive disorders, fluorosis, endocrine, thyroid and liver damages, and also decreases the growth hormone [1,2]. In addition, it influences the metabolism of some elements such as calcium and potassium [3]. Fluoride must be properly reduced before its discharge to the water bodies. Adsorption can be considered as an effective method for the removal of fluoride [4,5]. The applicability of P/γ-Fe_2_O_3_ nanoparticles for fluoride removal was reported. Fourier transform infrared (FTIR) on the P/γ-Fe_2_O_3_ nanoparticles is given in Fig. 1. Fig. 2 shows the schematic illustration for the synthesis of P/γ-Fe_2_O_3_ nanoparticles. The functional groups present in the P/γ-Fe_2_O_3_ nanoparticles before and after fluoride adsorption are given in Table 1. The estimated adsorption isotherm and kinetic parameters are presented in Table 2.

**Table 1:** Functional groups present in the P/γ-Fe_2_O_3_ nanoparticles before and after fluoride adsorption.

Peak (Absorbance) cm^−1^		Type of vibration or Bond source	Functional group name	Peak intensity description
Before adsorption	After adsorption			
481.62	550.60	C—I stretch	Alkyl halides	Strong
1637.16	1640.50	N—H bend	1° amines	Medium
2025.20	2077.11	—C≡C— stretch	Alkynes	Weak
3449.22	3452.39	O—H stretch, H— bonded	Alcohols and phenols	Strong and broad

**Table 2:** Isotherm and kinetic data for the sorption of fluoride on P/γ-Fe_2_O_3_ nanoparticles.

Isotherms	Freundlich			Langmuir			Temkin		
	*R^2^*	*K_f_*	*1/n*	*R^2^*	*q_m_*	*K_L_*	*R^2^*	*A_T_*	*B_1_*
C_0_(mg/L) 25	0.9	79.4	0.013	0.9999	81.3	0.012	0.995	1.19	1.088

Kinetics	Lagergren			Ho			Intraparticle diffusion		
	*R^2^*	*K_1_*	*q_e_*	*R^2^*	*K_2_*	*q_e_*	*R^2^*	*K_pi_*	*c*
C_0_(mg/L) 25	0.6262	0.005	1.7	0.999	0.067	100	0.78	0.0041	97.9

## Adsorption experiments

The adsorption experiment was conducted at batch mode using the one-factor-at-a-time (OFAT) method, that is, keeping a factor constant and varying the other factors to get the optimum condition of each variable. At first, for the purpose of this study, a stock solution of fluoride was prepared with distilled water from which other fluoride concentrations were prepared. The stock solution of fluoride (concentration of 1000 mg/L) was made by dissolving 2.21 g NaF in 1000 mL distilled water. A known mass of adsorbent (P/γ-Fe_2_O_3_ nanoparticles) was added to 1 L of the water samples containing different concentrations of fluoride. The pH of the water sample was adjusted by adding 0.1 N HCl or NaOH solutions. The removal efficiency was determined by varying the different adsorption process parameters such as pH (2–11), contact time (15–120 min), initial fluoride concentration (10–50 mg/L) and P/γ-Fe_2_O_3_ nanoparticles dosage (0.01–0.1 g/L). To create optimal conditions, the solutions were agitated using orbital shaker at a predetermined rate (150 rpm). After each experimental run, the solution was filtered and the filtrate was analyzed for the residual fluoride concentration. The initial and residual fluoride concentrations in the solutions were analyzed by a UV–vis recording spectrophotometer (Shimadzu Model: CE-1021-UK) at a wavelength of absorbance (λ_max_): 570 nm [5].

## Data analysis

The removal efficiency, *R* (%) and amount of fluoride adsorbed on P/γ-Fe_2_O_3_ nanoparticles, *q_e_* (mg/g) of the studied parameters were estimated based on the following formulas [[6], [7], [8]]:

(1) $$\%R=\frac{\left(C_{0}-C_{f}\right)}{C_{0}}100$$

Where *C_0_* and *C_f_* are the initial and residual fluoride concentrations (mg/g), respectively.

(2) $$q_{e}=(C_{0}-C_{e})\times \frac{V}{M}$$

Where *C_0_* and *C_e_* are the initial and final equilibrium liquid phase concentration of fluoride (mg/g), respectively. *M* is the weight of the nano adsorbent (g) and *V* is the volume of the solution (L).

## Influence of process variables

In this research, the influence of pH (2 - 11), contact time (15 - 120 min), initial fluoride concentration (15 - 50 mg/L) and P/γ-Fe_2_O_3_ nanoparticles dosage (0.01 - 0.1 g/L) on the removal efficiency was investigated.

**Fig. 3:**
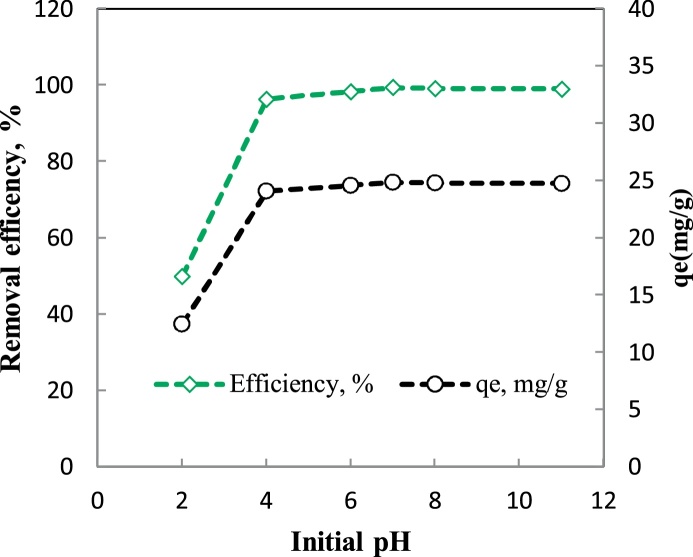
Effect of pH on the removal efficiency of fluoride on P/γ-Fe_2_O_3_ nanoparticles. (Contact time: 30 min, dosage: 0.09 g/L, initial fluoride concentration: 10 mg/L).

**Fig. 4:**
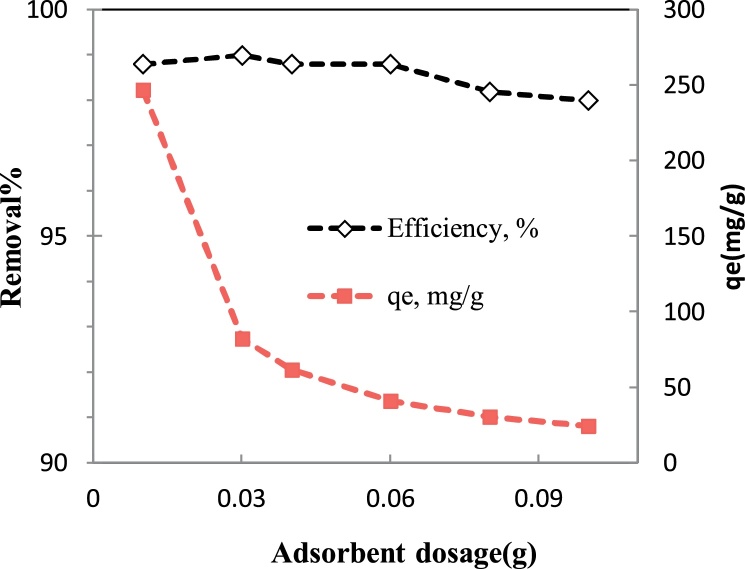
Effect of adsorbent dosage on the removal efficiency of fluoride. (Contact time: 30 min, optimum pH: 7, initial fluoride concentration: 10 mg/L).

**Fig. 5:**
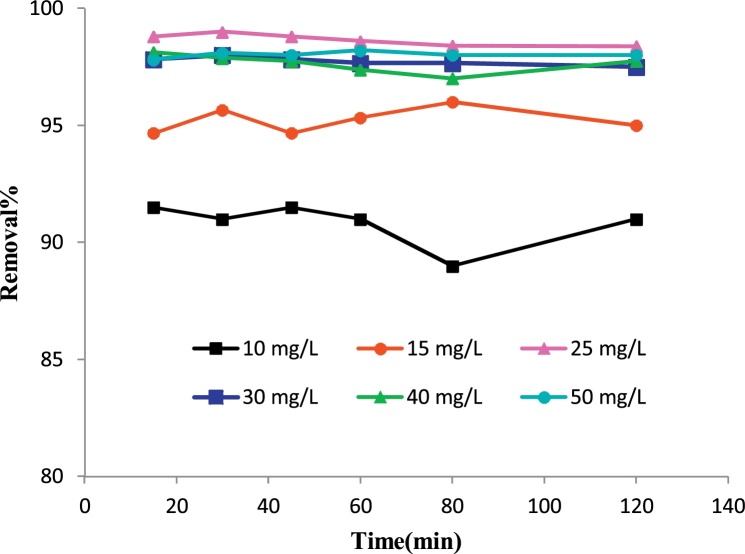
Effect of initial fluoride concentration on the removal efficiency of fluoride (optimum P/γ-Fe_2_O_3_ nanoparticles dosage: 0.02g/L, optimum pH: 7).

Higher removal efficiency was obtained at pH of 7 (Fig. 3), an adsorbent dosage of 0.02 g/L (Fig. 4), the initial fluoride concentration of 25 mg/L (Fig. 5) and contact time of 60 min (Fig. 5). This optimum conditions of pH 7, adsorbent dosage: 0.02 g/L, contact time: 30 min and initial fluoride concentration: 25 mg/L gave an efficiency of 99% (Fig. 5).

## Isotherm and kinetic modeling

An important physiochemical subject in terms of the evaluation of adsorption processes is the adsorption isotherm, which provides a relationship between the amount of fluoride adsorbed on the solid phase and the concentration of fluoride in the solution when both phases are in equilibrium [9]. To analyze the experimental data and describe the equilibrium status of the adsorption between solid and liquid phases, the Langmuir, Freundlich, and Temkin isotherm models were used to fit the adsorption isotherm data.

Several kinetic models have been applied to examine the controlling mechanisms of adsorption processes such as chemical reaction, diffusion control, and mass transfer [10]. Three kinetics models, namely pseudo-first-order, pseudo-second-order, and intraparticle diffusion models were used in this study to investigate the adsorption of fluoride on P/γ-Fe_2_O_3_ nanoparticles.

### Langmuir isotherm

For the Langmuir model, it is assumed that adsorbates attach to certain and similar sites on the adsorbent’s surface and the adsorption process occurs on the monolayer surface. The Langmuir equation can be rearranged to linear form for the convenience of plotting and determining the isotherm constants, *K_L_* and *q_m_* by drawing a curve of l/*q_e_* versus 1/*C_e_* [11,12]:

(3) $$\frac{{\text{C}}_{\text{e}}}{{\text{q}}_{\text{e}}}\;=\;\frac{{\text{C}}_{\text{e}}}{{\text{q}}_{\text{m}}}\;+\;\frac{1}{{\text{q}}_{\text{m}}{\text{K}}_{\text{L}}}$$

Where *q_e_* (mg/g) is the amount of fluoride adsorbed per specific amount of adsorbent, *C_e_* is the equilibrium concentration of the fluoride solution (mg/L), *K_L_* (L/mg) is Langmuir constant, and *q_m_* (mg/g) is the maximum amount of fluoride required to form a monolayer.

### Freundlich isotherm

The Freundlich model is an empirical relationship between the parameters, *q_e,_* and *C_e_*. It is obtained by assuming a heterogeneous surface with nonuniform distribution of the adsorption sites on the adsorbent surface, and can be expressed by the following equation [13,14]:

(4) $$q_{e}=\;K_{f}\;C_{e}^{\frac{1}{n}}$$

Where *K_f_* and *1/n* are the Freundlich constants related to adsorption capacity and adsorption intensity, respectively. The Freundlich constants can be obtained by plotting a graph of Log *q_e_* versus Log *C_e_* based on the experimental data by applying the linear form of the Freundlich isotherm Eq. (4):

(5) $$\log\left(q_{e}\right)=\log\left(K_{f}\right)+\frac{1}{n}\log\left(C_{e}\right)$$

### Temkin isotherm

In Temkin model, the surface adsorption theory was corrected considering possible reactions between the adsorbent and adsorbate. This model can be expressed as the following equation [15]:

(6) $$q_{e}=B_{1}\ln(A_{T})+B_{1}\ln(C_{e})$$

Where *A_T_* and *B_1_* are the Temkin constants. *B_1_* is related to the heat of adsorption and *A_T_* is the equilibrium binding constant.

### Lagergren kinetic model

Adsorption kinetic models are used to examine the rate of adsorption process and the potential rate controlling step. The Lagergren (pseudo-first-order) rate equation is expressed as Eq. (7) [16,17]:

(7) $$Log\left(q_{e}-q_{t}\right)=Log\left(q_{e}\right)-\frac{k_{1}}{2.303}t$$

### Ho kinetic model

The Ho (pseudo-second-order) rate equation is given as [12,18]:

(8) $$\frac{\text{t}}{{\text{q}}_{\text{t}}}\;=\;\frac{1}{{\text{K}}_{2}}\;+\;\frac{1}{{\text{q}}_{\text{e}}}\text{t}$$

Where *q_e_* (mg g^−1^) and *q_t_* (mg g^−1^) are the amounts of fluoride adsorbed at equilibrium and at time t, respectively, *K_1_* (min^−1^) is the pseudo-first-order rate constant of adsorption, and *K_2_* (g mg^−1^ min^−1^) is the pseudo-second-order rate constant.

### Intraparticle diffusion

For the intraparticle diffusion model (Eq. (9)), *c* is the intercept (mg/g) and *K_pi_* is the slope. If intraparticle diffusion is involved in the adsorption process, then the plot of *t_0.5_* versus *q_t_* would result in a linear relationship, and the intraparticle diffusion would be the controlling step if this line passed through the origin (C = 0). When the plots do not pass through the origin (C ≠ 0), this is indicative of some degree of boundary layer control and this further shows that the intraparticle diffusion is not the only rate controlling step, but also other processes may control the rate of adsorption [19,20].

(9) $$q_{t}=K_{pi}t^{0.5}+c$$

Where *q_t_* (mg/g) is the amount of fluoride adsorbed at time t (min) and *K_pi_* (mg/g min) is the intraparticle diffusion model rate constant.

**Fig. 6:**
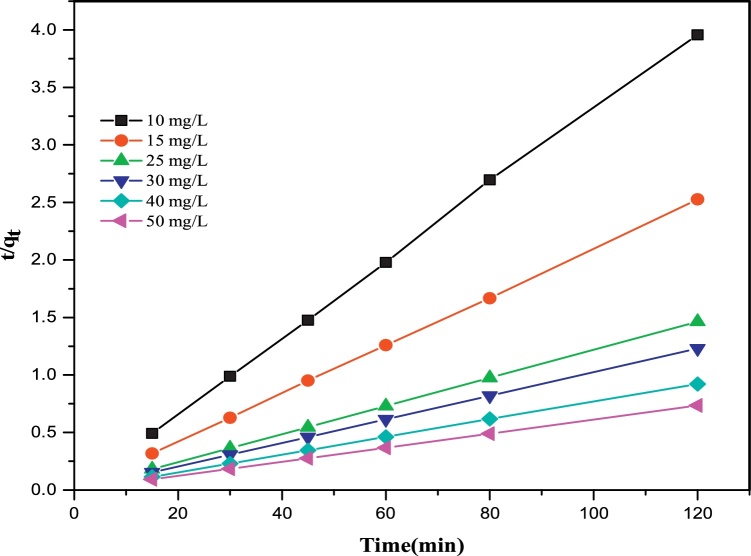
Pseudo-second-order (Ho) kinetic plot for fluoride removal on P/γ-Fe_2_O_3_ nanoparticles.

The estimated adsorption isotherm and kinetic parameter are presented in Table 2. Fig. 6 shows the adsorption kinetic (Ho) plot for fluoride removal on P/γ-Fe_2_O_3_ nanoparticles. The removal of fluoride on P/γ-Fe_2_O_3_ nanoparticles followed the Ho kinetic model with a correlation coefficient (*R^2^*) of 0.999 at 25 mg/L, suggesting that the rate-limiting step is a chemical adsorption process [21]. The isotherm data fitted into the Freundlich, Langmuir and Temkin isotherms but fitted more to the Langmuir isotherm, which indicates a monolayer adsorption on a homogeneous surface [14].

## Funding sources

This paper is the result of the approved project at Zabol University of Medical Sciences.
